# Esculeoside A Decreases Diabetic Cardiomyopathy in Streptozotocin-Treated Rats by Attenuating Oxidative Stress, Inflammation, Fibrosis, and Apoptosis: Impressive Role of Nrf2

**DOI:** 10.3390/medicina59101830

**Published:** 2023-10-14

**Authors:** Jozaa Z. ALTamimi, Nora A. AlFaris, Ghedeir M. Alshammari, Reham I. Alagal, Dalal H. Aljabryn, Mohammed Abdo Yahya

**Affiliations:** 1 Department of Physical Sports Sciences, College of Education, Princess Nourah bint Abdulrahman University, Riyadh 11671, Saudi Arabia; jzaltamimi@pnu.edu.sa (J.Z.A.); dhaljabryn@pnu.edu.sa (D.H.A.); 2 Department of Food Science and Nutrition, College of Food and Agricultural Sciences, King Saud University, Riyadh 11451, Saudi Arabia; aghedeir@ksu.edu.sa (G.M.A.); mabdo@ksu.edu.sa (M.A.Y.); 3 Department of Health Sciences, College of Health and Rehabilitation Sciences, Princess Nourah bint Abdulrahman University, Riyadh 11671, Saudi Arabia; rialagal@pnu.edu.sa

**Keywords:** Esculeoside A, diabetic, cardiomyopathy, oxidative stress, inflammation, Nrf2

## Abstract

*Background and Objectives:* This experiment evaluated the preventative influence of the tomato-derived Esculeoside A (ESA) on diabetic cardiomyopathy in type 1 diabetes mellitus (T1DM) in rats induced by streptozotocin (STZ). It also examined whether the activation of Nrf2 signaling affords this protection. *Materials and Methods:* Adult male Wistar control nondiabetic rats and rats with T1DM (STZ-T1DM) were given either carboxymethylcellulose as a vehicle or ESA (100 mg/kg) (eight rats/group) orally daily for 12 weeks. A group of STZ-T1DM rats was also treated with 100 mg/kg ESA and co-treated i.p. with 2 mg/kg (twice/week), brusatol, and Nrf2 inhibitors for 12 weeks. *Results and Conclusions:* Treatment with ESA prevented the gain in heart weight and cardiomyocyte hypertrophy and improved the left ventricular (LV) systolic and diastolic function (LV) in the STZ-T1DM rat group. Likewise, it reduced their serum levels of triglycerides, cholesterol, and low-density lipoproteins (LDL-c), as well as their LV mRNA, cytoplasmic total, and nuclear total levels of NF-κB. ESA also reduced the total levels of malondialdehyde, tumor necrosis factor-α, interleukine-6 (IL-6), Bax, cytochrome-c, and caspase-3 in the LV of the STZ-T1DM rats. In parallel, ESA enhanced the nuclear and cytoplasmic levels of Nrf2 and the levels of superoxide dismutase, glutathione, and heme oxygenase-1, but decreased the mRNA and cytoplasmic levels of keap-1 in the LVs of the STZ-T1DM rats. Interestingly, ESA did not affect the fasting insulin and glucose levels of the diabetic rats. All of these beneficially protective effects of ESA were not seen in the ESA-treated rats that received brusatol. In conclusion, ESA represses diabetic cardiomyopathy in STZ-diabetic hearts by activating the Nrf2/antioxidant/NF-κB axis.

## 1. Introduction

Type 1 diabetes mellitus (T1DM) is one of the most common metabolic disorders that is characterized by the absolute deficiency of insulin and hyperglycemia due to the autoimmune destruction of pancreatic beta (β) cells [1]. Globally, 8.4 million individuals are identified as having T1DM, a number that is projected to increase to between 13.5 and 17.4 million by 2040 [1]. The Kingdom of Saudi Arabia has the largest population in the Arabian Peninsula and accounts for about 33.3 million people [2]. Recent longitudinal and cross-sectional studies have shown that KSA occupies the 8th and 4th place in the world regarding the number of cases and incidence of T1DM, respectively, ranking it as one of the Arabian countries with the highest incidence rates of T1DM [2,3].

Diabetic cardiomyopathy is considered one of the greatest lethal complications of T1DM. It increases mortality rates among patients by altering the function of other organs and promoting heart failure (HF) [4]. At clinical levels, diabetic cardiomyopathy is characterized by severe systolic and diastolic dysfunction due to the increased death of cardiomyocytes, cardiomyocyte hypertrophy, and increased interstitial collagen deposition and fibrosis [5,6]. Oxidative stress is defined by an imbalance between pro-oxidants (reactive oxygen species (ROS)) and antioxidants. It is acknowledged as the main central pathway responsible for the development and progression of diabetic cardiomyopathy through promoting inflammation, hypertrophy, fibrosis, and apoptosis [4,7,8,9]. Hyperglycemia, hypoinsulinemia, neuro-hormonal activation, and microcirculation dysfunction are the major pathological mechanisms that trigger oxidative stress in diabetic hearts [5,10,11,12]. 

Nonetheless, the pathogenesis of diabetic cardiomyopathy is a complex mechanism that involves alterations in multiple interconnected signaling pathways such as the mitogen-activated protein kinase (MAPK), sirtuin-1 (SIRT1), AMP-activated protein kinase (MAPK), nuclear factor-kappa-beta (NF-κB), and the nuclear factor E2-related factor 2 (Nrf2) pathways [10]. Among these, accumulating data have shown that the Nrf2 signaling pathway is a major mechanism underlying the cardiac oxidative and inflammatory damaging effect of hyperglycemia in T1DM patients and animal models [13,14,15]. In general, Nrf2 is the major protective factor in the cell that inhibits cell apoptosis and enhances survival by suppressing oxidative stress and inflammation [14]. Under normal non-stressful situations, Nrf2 in the cytoplasm is tightly bound to the Kelch-like ECH-associated protein 1 (Keap1), which stimulates its degradation in the proteasomes [15]. However, ROS and electrophiles stimulate Nrf2 nuclear translocation via deactivating keap1 [15]. In the nucleus, Nrf2 binds the antioxidant response elements (AREs) to initiate the transcription of glutathione (GSH) and other endogenous phase II antioxidant enzymes [16]. The mRNA and Nrf2 activities are downregulated in the hearts of diabetic animals, as well as in cultured cardiomyocytes exposed to high glucose levels, and are positively correlated to the degree of apoptosis, inflammation, and fibrosis [13,17,18,19,20,21]. However, the activation of Nrf2 has preserved cardiac function and reduced cardiac oxidative, inflammatory, and apoptotic damage in these models. 

On the other hand, the consumption of plants confers protection against diabetic complications and is a major source for the discovery of some anti-diabetic drugs. These benefits are attributed to their valued ingredients, which act as potent antioxidant, hypoglycemic, hypolipidemic, and anti-inflammatory compounds [22,23]. In addition, several plant-derived compounds were reported to improve cardiac function and attenuate diabetic cardiomyopathy oxidative and inflammatory damage in both humans and animals [10,24]. Tomato is just one example of these medicinal plants that have a powerful potential to treat cancer, DM, cardiovascular disorders, hypertension, nephropathies, and skin disorders [25]. In these compounds, tomato is rich in several protective compounds such as lycopene, α-tomatine, resveratrol, kaempferol, vitamin C, and phenolic acids [25]. Esculeoside A (ESA) is a newly discovered spirosolane steroidal glycoside from tomato [26]. ESA can be found in ripe red tomato fruits, where it is produced from the oxidation of tomatine, another compound that is more dominant in green fruits [27]. The pharmacological properties of ESA were not studied well. The available data have depicted some potentials of ESA to act as a hypoglycemic, insulin-sensitizing, anti-allergic, antioxidant, anti-inflammatory, and hypolipidemic molecule [26,27,28,29]. 

However, the cardioprotective effect of ESA has never been tested before in diabetic animals or any other model of cardiovascular dysfunction. Therefore, the present investigation was conducted with two major goals. The first goal was to examine whether the chronic administration of ESA can alleviate cardiac injury and dysfunction in streptozotocin (STZ)-diabetic rats. The second goal was to determine the possible mechanisms of action, including targeting glucose and lipid hemostasis, as well as cardiac endogenous mechanisms such as Nrf2/antioxidant and inflammatory signaling pathways. 

## 2. Materials and Methods

### 2.1. Animals

All rats utilized in the current work were 7–9-week-old adult Wistar male rats weighing 120–136 g. These rats were obtained from the Experimental Animal Care Center at King Saud University, Saudi Arabia. Four animals were housed per cage at a temperature of 21 °C under equal light/dark phases (12 h). During the adaptation and experimental period, the animals were nourished using a normal standard chow diet. Water was supplied ad libitum. The provided experimental protocol was appraised and approved by the animal committee at King Saud University (IRB Number 20-0096).

### 2.2. Establishment of the T1DM Model

Streptozotocin (STZ) was used to induce T1DM in the rats. The drug was purchased as a powder from Sigma (Cat. number (S0130), Sigma Aldrich, St. Louis, MO, USA) and freshly prepared in 0.1% citrate buffer. Each rat was injected with 55 mg/kg of STZ solution intraperitoneally. This dose improves cardiac function and promotes cardiac injury in rats, 3 days post-injection, by promoting inflammation and oxidative stress [30]. Simultaneously, other rats were administered the vehicle (buffer) and were considered controls. Three days later, fasting blood glucose was monitored using a tail blood sample from both groups using an Active glucometer (Accu-Chek) (Roche Diagnostics GmbH, Mannheim, Germany). The cutoff value for the establishment of DM was set at 300 mg/kg. 

### 2.3. Preparing ESA 

The pure extract of ESA was prepared from fresh ripe tomatoes following previous studies [31]. Briefly, fresh local ripe tomatoes were purchased from the vegetable market of Riyadh, Saudi Arabia, during the cultivation period. The fruits were washed with water and then smashed and filtered. The filtrate was centrifuged at low speed for 10 min (500× *g*/room temperature), and the supernatants were isolated. The ESA was isolated using column chromatography with a Diaion HP20 (Cat. Number 13616, Aldrich, St. Louis, MO, USA). Elution was performed with 60% methanol and through reverse silica gel columns. The organic layer was removed under pressure, and the pellet containing the ESA was identified by the staff of the College of Pharmacy at our institution. This protocol yielded 0.057% of ESA, and the structure of the ESA was similar to the above-mentioned previous reports. At the time of use, the ESA pellet was freshly dissolved in 0.5% carboxymethyl cellulose (CMC).

### 2.4. Experimental Groups

The diabetic and healthy rats were divided into five groups (n = 8 each/total 40 rats). These included control + CMC (vehicle)-treated rats, control + ESA (100 mg/kg), STZ model, STZ + ESA (100 mg/kg of body weight (bw)), and STZ + ESA + brusatol (an Nrf2 inhibitor, 2 mg/kg). The utilized dose of ESA showed hypoglycemic and hypolipidemic effects in mice and rats [28,31]. We previously tested and used brusatol to suppress Nrf2 activities in vivo in rats [32]. Treatment with ESA was administered orally via a gavage feeding needle daily. Two doses of brusatol were given per week [32]. All treatments were terminated after 84 days, the time at which diabetic cardiomyopathy was induced in the rats at the selected dose of STZ [30]. 

### 2.5. Assessment of LV Function

The assessment of the left ventricular (LV) function was carried out as previously shown in our prior studies [32]. During week 12, the rats were anesthetized as described by Rakhshandeh et al. [33] using ketamine (85 mg), acepromazine (3 mg/kg), and xylazine (8 mg/kg, *v*/*v*) [34]. In brief, the carotid artery was localized and freed from the vagus nerve. LV function was recorded by inserting a pressure Millar catheter into the LV through the carotid artery. The LV pressure signal was recorded using a PowerLab data question system (Version 8, ADInstruments, New South Wales, Australia). The first 5 min of the recording was ignored. The following parameters were derived from the recorded pressure signal: LV systolic and the end-diastolic pressures (LVSP/LVEDP) and the maximum rise/reduction in pressure over time (dp/dtmax or dp/dtmin). 

### 2.6. Assessment of Biochemical Parameters in the Plasma and Serum 

At the end of the experiment, and after a 12 12-h fasting period, the blood samples of the rats were collected directly from their heart. Serum and plasma were prepared from all samples (500× *g*/10 min/room temperature) and preserved at −20 °C. Fasting glucose and insulin in the plasma were determined using ELISA colorimetric kits (Cat. Number 10009582, Cayman Chemicals, CA, USA, and Cat. Number ERINS, ThermoFisher, Waltham, MA, USA). The levels of all lipids, including triglycerides (TG), cholesterol (CHOL), low-density lipoprotein cholesterol (LDL-c), lactate dehydrogenase (LDH), troponin I (TPI), and creatinine kinase-MB (CK-MB), of the serum samples were assessed using an automated auto-analyzer (model BA-A-120, Bioevopeak INC, Jinan City, China). All protocols were conducted as provided by the manufacturer of each kit. The analysis was carried out in duplicate for n = 8 samples/group. The analysis of serum brain natriuretic peptide (BNP) and atrial natriuretic peptide (ANP) was conducted using ELISA (Cat. number MBS2513078 and Cat. number MBS2503794, MyBioSorces, San Diego, CA, USA). 

### 2.7. Analyses of the Biochemical Markers in the Tissues 

The experimental rats were ethically killed by dislocation of their necks. The heart of each rat was isolated on ice and weighed. The LV of each heart was separated, snap-frozen in liquid nitrogen, and then preserved at −70 °C. Homogenates from these LVs were prepared using neutral phosphate-buffered saline. The nuclear and cytoplasmic fractions of the LVs were extracted using a commercial kit (Cat. number NT-032; Invent Biotechnologies, Plymouth, MN, USA). Rat-specific ELISA kits were used to measure the cardiac content of malondialdehyde (MDA), intracellular cell adhesion molecule (ICAM-1), interleukin-6 (IL-6), tumor necrosis factor-alpha (TNF-α), heme oxygenase-1 (HO-1), total GSH, and superoxide dismutase-1 (Cu/Zn; SOD1) (Cat. number MBS269892; MyBioSorces, San Diego, CA, USA; Cat. Number Ab100763, Abcam, Trumpington, Cambridge, UK; Cat. number MBS9501941, MyBioSorces, San Diego, CA, USA; Cat. number K739-100, Biovision, TX, USA; Cat. number ADI-EKS-810A; ENZO, Aurora, IL, USA; Cat. number E1444Ra, Bioassay Technology Laboratory, Jiaxing, China; and Cat. Number RTEB1811, AssayGenie, Dublin, Ireland). The total levels of Type-1 collagen and transforming growth factor-beta1 (TGF-β1) in all LV samples were assessed using ELISA (Cat. number 6013, Chondrex, Woodinville, WA, USA and Cat. number MBS824788, MyBioSorces, San Diego, CA, USA). NF-κb p65, keap1, and Nrf2 in the cytoplasmic and nuclear fractions were assessed through ELISA (Cat. number CSB-E13148r, CUSABIO, Houston, TX, USA, Cat. number MBS7218529, MyBioSorces, San Diego, CA, USA; Cat. number NBP3-08161, Novus biologicals, Centennial, CO, USA). The levels of the B-cell lymphoma/leukemia 2 (Bcl2), cytochrome-c, and Bcl2-associated X protein (Bax) were also measured using ELISA (Cat. Number E-EL-R0096, Cat. Number E-EL-R0098; Cat. Number E-EL-R0006; and Cat. Number E-EL-R0160, Elabscience, Houston, TX, USA). Each experiment was implemented in duplicate for n = 8 samples/group as per the manufacturer’s recommendation. 

### 2.8. Real-Time Polymerase Chain Reaction (qPCR)

The primers used to measure the transcription of Nrf2, NF-κB, keap1, and β-actin (reference gene) are shown in Table 1. The reagent TRIzol was utilized as per instruction for the total RNA isolation from the frozen LVs. The isolated RNA was freshly treated with DNase to remove precipitated DNA, and then the sample clarity was determined using a nanodrop spectrophotometer at 260/280 nm. The synthesizing of the first-strand cDNA was carried out using the RevertAid First Strand cDNA Synthesis Kit (Cat. # K1621, ThermoFisher Scientific, Waltham, MA, USA). qPCR was accomplished using the Ssofast Evergreen Supermix kit (Cat. # 172-5200, Bio-Rad, Hercules, CA, USA) with CFX95 according to the steps established by the supplier. All amplifications were performed as n = 8 samples/group and were performed in duplicate.

### 2.9. Histology Studies

We evaluated the collagen depositions and the alterations in the structure of the LVs from all samples using Masson trichrome and hematoxylin and eosin (H&E) staining, respectively, as routinely and previously shown in our laboratories [35]. Briefly, freshly collected samples (1 mm^3^) of the LVs of all groups were preserved in buffered formalin 10% for 30 h. Further treatments included rehydration in ethanol, clearance in Xyline, and embedding in paraffin. Sections of 3 µm were prepared using a rotary microtome. All samples were observed by a blind evaluator, and images were captured at 200× under a light microscope. 

### 2.10. Statistical Analysis

All data were analyzed with GraphPad Prism software (Sydney/version 8). The normality was tested using the Kolmogorov–Smirnov test. The significance differences were determined by one-way ANOVA and Tukey’s’ post hoc test. The significance difference was determined at the level *p* < 0.05. All resulting values were presented as means ± SD.

## 3. Results

### 3.1. ESA Increases Body Weight, Reduces Heart Weight, and Improves LV Function in STZ-T1DM-Treated Rats by Stimulating Nrf2

The heart weights, LVEDP, and serum levels of TPI, CK-MB, and LDH were significantly higher, but the final body weights, cardiac LVSP, and max/min dp/dt were significantly lower in the STZ-T1DM diabetic rats compared with those of the control group (Table 2). The heart weights, cardiac levels of LVEDP, and serum concentrations of TPI, CK-MB, and LDH decreased with significant differences at *p* < 0.05, but the final body weights and cardiac levels of LVSP and max/min dp/dt were significantly augmented in the STZ-T1DM + ESA-treated rats (Table 2). This was reversed in the STZ-T1DM + ESA + brusatol-treated rats compared to STZ + ESA-treated ones (Table 2). None of these marker levels showed any significant differences between the control and ESA-treated rats or the STZ-T1DM + ESA and STZ-T1DM + ESA + brusatol-treated rats (Table 2).

### 3.2. ESA Fails to Improve Glucose and Insulin Levels but Attenuates Dyslipidemia in STZ-T1DM Rats through an Nrf2-Dependent Mechanism

Fasting plasma glucose and serum CHOL, TGs, and LDL-c significantly increased, while fasting insulin was depressed significantly in the STZ-T1DM-treated animals from that of the controls (Table 3). No significant alterations in fasting glucose were seen among control and ESA-treated rats or the STZ-T1DM, STZ-T1DM + ESA, and STZ-T1DM + ESA + brusatol-treated animals (Table 3). However, lower levels of CHOL, TGs, and LDL-c were seen in the serum of ESA-treated rats and STZ-T1DM + ESA-treated rats compared to the control group or STZ-T1DM model rats, respectively (Table 3). The serum levels of all of these lipid markers were significantly higher in the STZ-T1DM + ESA + brusatol-treated rats than in the STZ-T1DM + ESA-treated group, but did not differ significantly when compared to the STZ-T1DM diabetic rats (Table 3).

### 3.3. ESA Reduces Lipid Peroxidation and Inflammatory Cytokine Production and Increases Antioxidant Levels in the LVs STZ-T1DM Rats in an Nrf2-Dependent Mechanism

The LVs of SOD1, HO-1, and GSH were advanced significantly in the ESA-treated rats when compared with the control rats (Table 4). There was an increment in the levels of TNF-α, ICAM-1, MDA, and IL-6 with significant differences with a parallel lessening in the GSH, HO-1, and SOD1 levels in the LVs of the STZ-T1DM rats compared to the controls (Table 4). All of these marker levels were significantly inverted again in the LVs of the STZ-T1DM + ESA-treated rats when compared to the STZ-T1DM-treated rats (Table 4). The LVs of the STZ-T1DM + ESA + brusatol-treated rats showed higher concentrations of TNF-α, ICAM-1, MDA, and IL-6 and lower levels of GSH, HO-1, and SOD1 compared to the controls and STZ-T1DM + ESA-treated rats (Table 4). The values of each of these chemical endpoints were not statistically different amongst the STZ-T1DM rats and the STZ-T1DM + ESA + brusatol-treated group (Table 4).

### 3.4. ESA Reduces Cytoplasmic and Nuclear Levels of Nrf2 by Increasing the Cytoplasmic Levels of kepa-1 in the LVs of STZ-T1DM Rats

**Figure 1:**
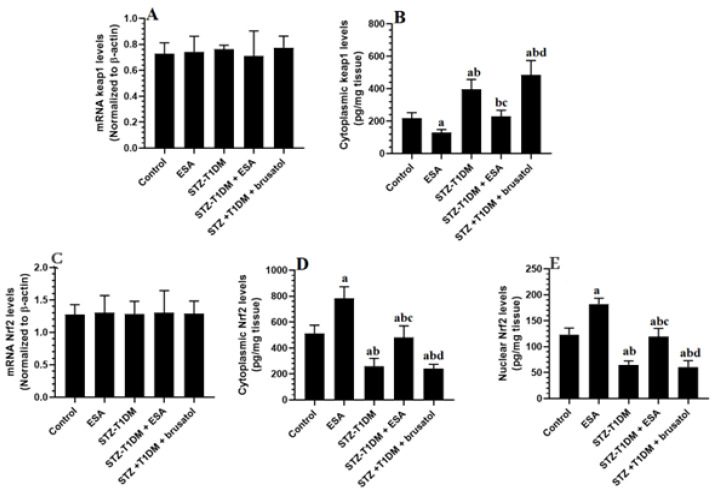
mRNA levels of keap1 (**A**), cytoplasmic levels of keap1 (**B**), mRNA levels of Nrf2 (**C**), cytoplasmic levels of Nrf2 (**D**), nuclear levels of Nrf2 (**E**), and nuclear levels of Nrf2 in the LVs of all groups of rats. Data are presented as mean ± SD. Level of significance = *p* < 0.05. a: significant vs. control group. b: significant vs. ESA (100 mg/kg)-treated rats, c: significant vs. STZ-T1DM model group. d: significant vs. different STZ-T1DM + ESA (100 mg/kg)-treated rats. ESA: Esculeoside A (ESA). Brusatol: an Nrf2 inhibitor.

The Nuclear Levels of keap1 and Nrf2 did not show significant differences among the study groups. Nevertheless, the cytoplasmic levels of keap1 increased with significant differences, while levels of Nrf2 declined significantly in the LVs of the STZ-T1DM rats compared to the controls (Figure 1A–E). The cytoplasmic and nuclear levels of Nrf2 were significantly higher, while the cytoplasmic levels of keap1 showed a significant reduction in the LVs of ESA- and STZ-T1DM + ESA-treated animals in comparison with the control group or STZ-T1DM model rats (Figure 1A–E). These data suggest that ESA stimulates the cytoplasmic availability and the transactivation of Nrf2 by increasing the degradation of kepa1 in the cytoplasm (Figure 1A–E). However, the STZ-T1DM + ESA + brusatol-treated rats had significantly higher cytoplasmic levels of keap1 and significantly lower cytoplasmic and nuclear levels of Nrf2 compared to the STZ-T1DM + ESA-treated rats—levels that were not significantly different compared to the STZ-T1DM model rats (Figure 1A–E). 

### 3.5. ESA Suppresses the Transcription and the NF-κB Activation by Activating Nrf2

**Figure 2:**
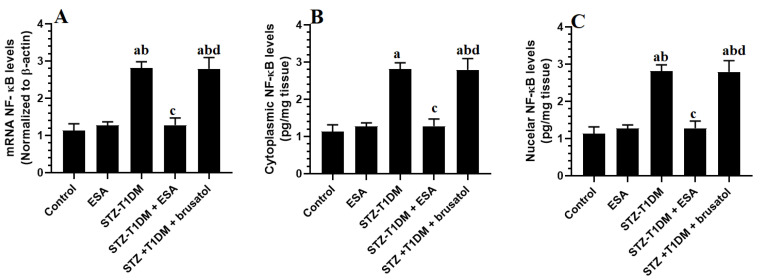
mRNA levels of NF-κB (**A**), cytoplasmic levels of NF-κB (**B**), and nuclear levels of NF-κB (**C**) in the LVs of all groups of rats. Data are presented as mean ± SD. Level of significance = *p* < 0.05. a: significant vs. control group. b: significant vs. ESA (100 mg/kg)-treated rats, c: significant vs. STZ-T1DM model group. d: significant vs. different STZ-T1DM + ESA (100 mg/kg)-treated rats. ESA: Esculeoside A (ESA). Brusatol: an Nrf2 inhibitor.

The mRNA levels, along with the nuclear and cytoplasmic NF-κB values, were significantly stimulated in the LVs of the STZ-T1DM model rats in comparison with the control rats (Figure 2A–C). The mRNA levels either in the cytoplasmic or nuclear levels of NF-κB of the control and ESA-treated rats did not differ significantly (Figure 2A–C). However, lower mRNA levels and reduced nuclear and cytoplasmic NF-κB were observed in the LVs of the STZ-T1DM rats compared to the STZ-T1DM model rats and the STZ-T1DM + ESA + brusatol-treated rats (Figure 2A–C). No significant differences in mRNA or nuclear and cytoplasmic NF-κB levels appeared among the STZ-T1DM model rats or STZ-T1DM + ESA + brusatol-treated rats (Figure 2A–C).

### 3.6. ESA Inhibits Intrinsic Cell Apoptosis in LVs by Stimulating Nrf2

**Figure 3:**
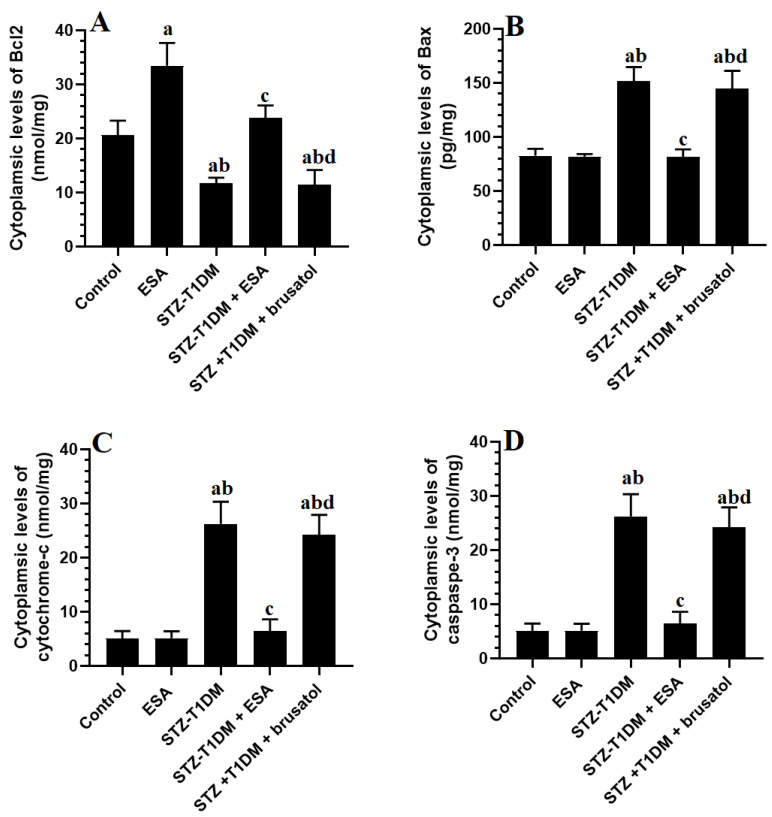
Cytoplasmic levels of Bcl2 (**A**), Bax (**B**), cytochrome-c (**C**), and caspase-3 (**D**) in the LVs of all groups of rats. Data are presented as mean ± SD. Level of significance = *p* < 0.05. a: significant vs. control group. b: significant vs. ESA (100 mg/kg)-treated rats, c: significant vs. STZ-T1DM model group. d: significant vs. different STZ-T1DM + ESA (100 mg/kg)-treated rats. ESA: Esculeoside A (ESA). Brusatol: an Nrf2 inhibitor.

The LV levels of cytoplasmic cytochrome-c, Bax, Bcl2, and caspase-3 did not differ significantly between the control and ESA-treated rats (Figure 3A–D). While the cytoplasmic levels of Bcl2 showed a significant reduction, the cytoplasmic Bax, cytochrome-c, and cleaved caspase-3 were higher with significant differences in the LVs of the STZ-T1DM and STZ-T1DM + bursal-treated group compared to the control rats, and were significantly reversed in the LVs of the STZ-T1DM + ESA-treated rats (Figure 3A–D). No significant difference was observed between these parameter levels found among the STZ + ESA + brusatol-treated rats and STZ-T1DM model rats (Figure 3A–D).

### 3.7. ESA Improves the Structure of the Cardiomyocytes of the LVs of STZ-T1DM

**Figure 4:**
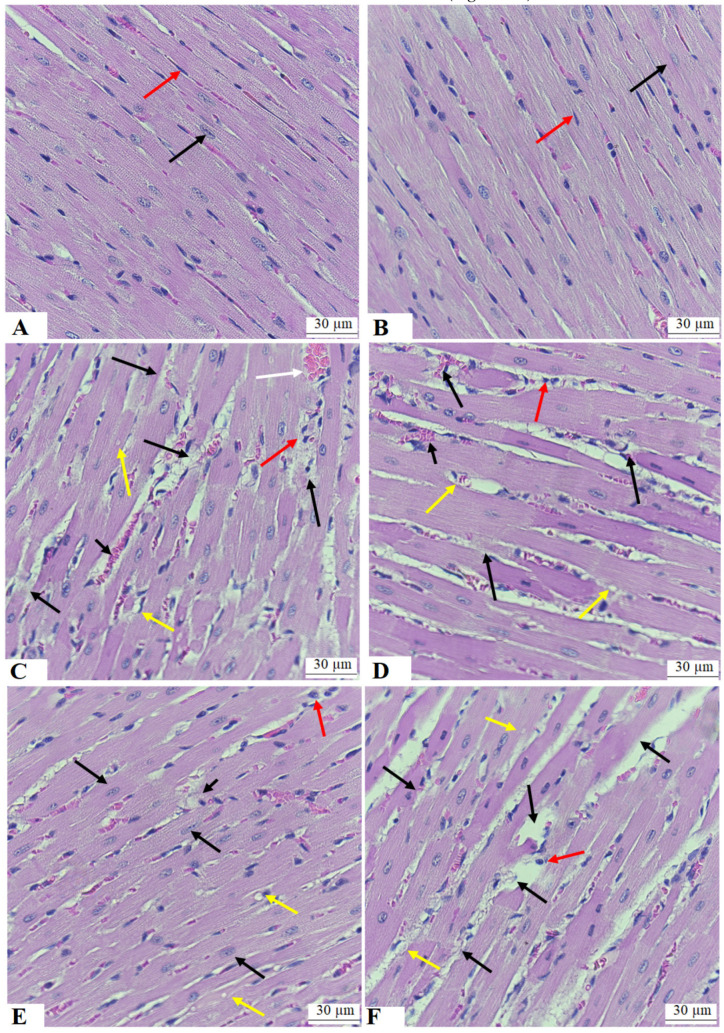
Histological images of the left ventricles (LVs) from all experimental groups of rats. (**A**,**B**) were taken from the control and ESA-treated rats and showed normal cardiomyocyte structure, with normal striation oval central nuclei (black arrow) and fibroblast running between the cardiomyocytes (red arrow). (**C**) was taken from T1DM-STZ rats and showed increased damage (black arrow) and vacuolization (yellow arrow) in the cardiomyocytes with dilated blood vessels (white arrow) and immune cell infiltration (red arrow). Note the increased volume in the size of the cardiomyocytes in these images. (**D**) was taken from an STZ-T1DM + ESA-treated rat and showed much improvement in the structure of the LVs, where most of the cardiomyocytes appeared normal and had normal-sized and oval central nuclei (black arrow). However, some vacuoles were still seen in the myocardium (yellow arrow). (**E**) was taken from an STZ-T1DM + ESA + brusatol-treated rat and showed similar pathological changes to those seen in the LVs of the STZ-T1DM model rats (image (**F**)).

Normal cardiomyocyte structures showing normal striation, oval centrally located nuclei, endothelial cells, and blood vessels were seen in the LVs of both the control and ESA-treated LVs (Figure 4A,B). Increased cardiomyocyte damage, vacuolization, hypertrophy with dilated blood vessels, and increased immune cell infiltrations were seen in the LVs of the STZ-T1DM-treated rats and the STZ-T1DM + ESA + brusatol-treated rats (Figure 4C,E). Almost normal cardiomyocyte structures with very little damage in the cardiomyocytes and little vacuolization were observed in the LVs of the STZ + ESA-treated rats (Figure 4D).

## 4. Discussion

**Figure 5:**
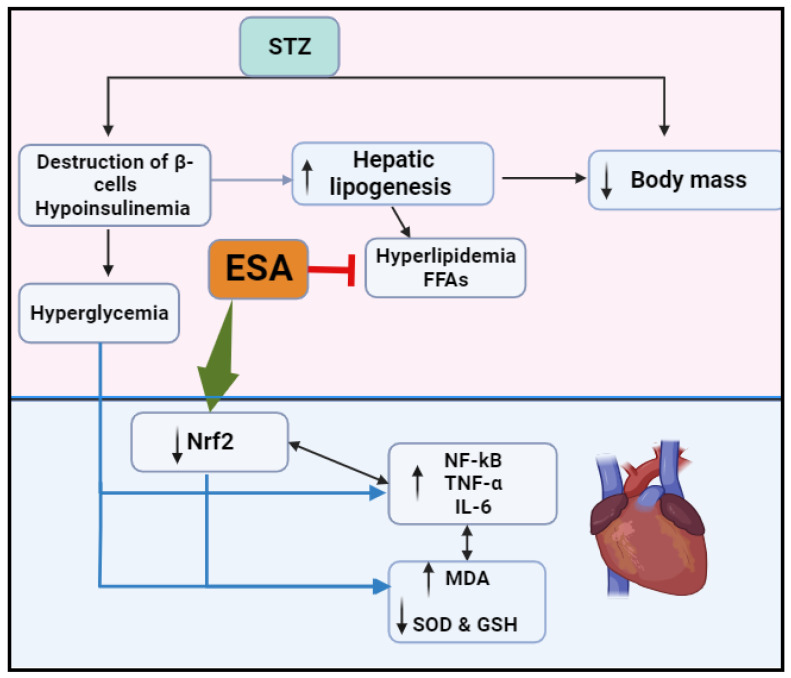
Graphical abstract demonstrating the role of Esculeoside A in attenuating cardiac damage in STZ-diabetic rats. This includes the activation of the Nrf2/NF-κB/antioxidant axis.

Despite the development and use of glucose-, lipid-, and hypertension-lowering drugs, the incidence of diabetic cardiomyopathy and associated deaths is still increasing. This reflects the lack of novel drugs that effectively act on key molecular mechanisms. Previous studies have shown the importance of the Nrf2/antioxidant axis in the prevention of diabetic cardiomyopathy, even in the presence of hyperglycemia. In this study, we provide the first evidence in the literature for the exceptional potential of ESA to alleviate diabetic cardiomyopathy by improving the impairment in cardiac function, preventing cardiomyocyte damage, and attenuating oxidative stress, inflammation, fibrosis, and apoptosis (Figure 5). However, this cardioprotection does not involve modulating glucose and insulin levels, but seems to involve at least a hypolipidemic effect and the cardiac-specific activation of Nrf2. In support, co-treatment with brusatol, a selective Nrf2 inhibitor, completely abolished all of the cardiac protective effects of ESA and exaggerated oxidative and inflammatory cardiac damage. 

The STZ-treated animal model is the best-described experimental model to induce T1DM [36]. STZ can selectively penetrate the pancreatic β-cells and destroy them by damaging their DNA [36]. Like the symptoms seen in humans, the clinical features of STZ-diabetic rats include severe and sustained hyperglycemia, hypoinsulinemia, polyphagia, a reduction in weight loss, polyuria, and dyslipidemia [30,36]. In addition, STZ-induced DM impairs cardiac systolic and diastolic function, damages cardiomyocytes in rodents, and results in HF by promoting hypertrophy, fibrosis, and apoptosis, reducing contractility, and impairing diastolic and filling time [21,37,38]. Hyperglycemia and insulin deficiency, in this model, remain the two major key events responsible for cardiac damage during the development and progression of T1DM [10]. Both factors generate large quantities of ROS and promote lipotoxicity in the heart by shifting the cardiac metabolism toward a reduction in glucose utilization and increasing the uptake and oxidation of FFAs [39,40]. In addition, hyperlipidemia is considered an independent factor for ischemic heart disease [40]. It can also facilitate the progression of HF in diabetic hearts by promoting lipotoxicity and the generation of ROS [40]. ANP and BNP are two major HF markers released into the serum from the hypertrophied cardiac wall stain [41,42]. In addition, LDH, CK-MB, and TPI are important markers for assessing cardiomyocyte injury, as they are released from the damaged cardiomyocytes and are markers of cell membrane disintegration [43]. 

Consistent with the studies mentioned above, the STZ-diabetic rats in this study developed hyperglycemia, hypoinsulinemia, and hyperlipidemia and suffered from a loss of body weight, all of which validate this T1DM animal model. We have also confirmed the diabetic cardiomyopathy in these rats by the increment in heart weights, impairments in cardiac systolic and diastolic function, the morphological damage of the cardiomyocytes, increased collagen synthesis and deposition, and higher serum levels of TPI, LDH, CK-MB, ANP, and BNP. On the other hand, we have also found that ESA treatment can ameliorate these listed features of diabetic cardiomyopathy, but with no alteration in insulin or glucose plasma levels. ESA was also able to reduce circulatory levels of TGs, CHOL, LDL-c, and FAs. Such findings indicate that ESA’s cardioprotective effect is not mediated by lowering glucose levels or improving insulin secretion, but involves a further hypolipidemic effect that seems concomitant with other cardiac local cellular mechanisms, as discussed later. 

However, the ability of ESA to improve diabetic rats’ body weight, even in the absence of insulin, could be explained by the protective effect of ESA on hyperglycemia-mediated toxicity and cell death in other tissues. It could also be explained by the significant increase in fat and muscle mass, which could be due to enhancement in peripheral insulin action. This can be supported by the study of Yang et al. [26], who showed the ability of ESA to improve peripheral insulin sensitivity in dp/dp by stimulating the AMPK-induced insulin sensitivity in the muscles [26]. Unfortunately, we did not measure fat and muscle weight, nor did we study the AMPK activities in these tissues to confirm such a suggestion. This will be considered in future studies. On the other hand, ESA also reduced circulatory levels of TGs, CHOL, and LDL-c in the serum of control rats, suggesting potent hypolipidemic effects in steady and diabetic conditions. These data support the previous results reported by Fujiwara et al. [28] and Yang et al. [26], who confirmed that ESA has a potent hypolipidemic effect in ApoE-deficient mice through downregulating and suppressing the intestinal and hepatic acyl-coenzymeA (CoA): cholesterol acyl-transferase (ACAT)-1 or ACAT-2. 

Nonetheless, the heart has low levels of endogenous antioxidants compared to other organs, making it highly vulnerable to oxidative damage [44]. Oxidative stress is the most well-known upstream cardiac mechanism that initiates hypertrophy, inflammation, and remodeling during the development and progression of diabetic cardiomyopathy, all of which ultimately end with cell death [8,9,11,45]. In diabetic hearts, and due to increasing FA utilization, the mitochondria remain the major source of ROS [11,45]. Other resources of ROS include the hyperglycemia-derived formation of advanced glycation end products (AGEs) and the activation of nicotinamide adenine dinucleotide phosphate oxidase (NADPH oxidase), protein kinase C (PKC), and NO synthases (NOS) [45]. In turn, ROS damage the cardiomyocytes by scavenging the cellular antioxidants and promoting lipid peroxidation, damaging the DNA, mitochondria, and membranes, inducing protein oxidation and carbonylation, inflammation, fibrosis, and apoptosis [9]. In addition, ROS and AGEs reduce cardiac contractility and induce cardiac stiffness by impairing Ca^2+^ hemostasis through altering SERCA and Na^+^/Ca^2+^ ATP exchangers [46,47]. ROS and AGEs can also stimulate cardiac inflammation by activating NF-κB, a major transcription factor responsible for synthesizing and expressing adhesive molecules and inflammatory cytokines [48]. ROS and inflammatory cytokines, such as IL-6 and TNF, lead to cardiac fibrosis and stiffness through upregulating TGF-β1, collagen synthesis, and stimulating the matrix metalloproteinases (MMP) [48,49]. 

The oxidative stress response in the LVs of STZ-diabetic hearts in this study was characterized by the increased production of lipid peroxides and reduced levels of GSH, SOD, and HO-1. In addition, the data of this study also confirmed inflammation in the LVs of these rats by the significant upregulation in the mRNA levels and nuclear levels of NF-κB and higher levels of ICAM-1, TNF-α, IL-6, and IL-1β. These data follow many other studies that have also shown similar results on the reduction in these antioxidant levels and in the elevation of MDA, TNF-α, IL-6, and collagen levels, as well as the expression/activation of NF-κB in the hearts of STZ-diabetic rats [32,50,51,52,53,54,55,56,57]. However, restoring all of these biochemical endpoints in the LVs of these rats was our strongest evidence that the cardioprotective effect of ESA is mediated by suppressing oxidative stress, inflammation, and fibrosis. In addition, treatment with ESA also stimulated the levels of all antioxidants in the LVs of the control rats without altering the expression and activity of NF-κB or the levels of collagen and other inflammatory cytokines. These data suggest that ESA mainly acts to upregulate antioxidants and confer that the anti-inflammatory effects of ESA are secondary to its antioxidant potential. These data are novel in showing such antioxidant and anti-inflammatory potentials of ESA, which were not investigated enough in the literature. However, the study of concanavalin A (ConA)-blast mouse splenocyte primary culture cells [31] has suggested that the anti-inflammatory effect of ESA is mediated by suppressing CD4+ T-cells. 

On the other hand, three forms of cell death were described in diabetic hearts and are involved in the pathogenesis of diabetic cardiomyopathy. These include apoptosis, necrosis, and autophagy [10,58]. Apoptosis is a programmed cell death classified as intrinsic and extrinsic cell death modalities in extrinsic or intrinsic mechanisms [58]. TNF-α is a major contributor that triggers the extrinsic pathway of cell apoptosis by binding to death receptors [59]. On the other hand, intrinsic cell death results from the adverse effect of ROS, inflammatory cytokines, disturbances in Ca^2+^ levels, and DNA damage [55,60,61]. Also, intrinsic cell apoptosis is characterized by the reduced expression of anti-apoptotic genes (e.g., Bcl2), the overexpression of apoptotic genes such as Bax, damaged mitochondria, and increased mitochondrial cytochrome-c release, which ultimately activates caspase-3 and caspase-9, fragmenting the DNA [62]. Both extrinsic and intrinsic cell death modality was observed in the diabetic hearts of STZ-treated animals, as well as in the LV biopsies of patients with T1DM, and was attributed to oxidative damage [55,57,58,63]. Intrinsic cell apoptosis was also confirmed in the LVs of the STZ-diabetic animals in this study, as shown by their high cytoplasmic content of cytochrome-c, Bax, and caspase-3 and the lower levels of Bcl2.

Interestingly, ESA alleviated cardiac apoptosis by decreasing the ventricular content of Bax, cytochrome-c, and caspase-3 and increasing the content of Bcl2. Such an effect of ESA could be explained by the ability of ESA to reduce levels of ROS, TNF-α, and IL-6 and inhibit DNA damage. On the other hand, we observed an exceptional ability of ESA to stimulate Bcl2 in the levels of the control rats, too, suggesting a stimulatory role in the expression of anti-apoptotic genes. These data suggest that ESA may stimulate cell survival and resistance to oxidative/inflammatory-induced death by boosting Bcl2 in diseased and healthy myocardia. 

Our final aim is to understand the possible mechanisms responsible for the observed antioxidant and anti-apoptotic effects of ESA. Therefore, we targeted Nrf2 due to its role in stimulating the synthesis of GSH and antioxidant enzymes [15]. In addition, Nrf2 is a key factor that stimulates the expression of Bcl2 [64]. Furthermore, Nrf2 can directly inhibit NF-κB by the phosphorylation-induced suppression of IKK, which normally activates NF-κB [65,66]. It also suppresses collagen deposition and attenuates fibrosis by reducing the expression of TGF-β1 [67]. In addition, we treated the STZ-diabetic rats with ESA administered with brusatol, a known inhibitor that stimulates the cytoplasmic degradation of Nrf2 and decreases its nuclear translocation. As expected, the total cytoplasmic and nuclear levels of Nrf2 were significantly depleted in the LVs of the STZ-diabetic rats, but with no alteration in the mRNA levels. This could be due to higher apoptotic rates in these cells or the increased cytoplasmic degradation of Nrf2. Supporting this, we also observed a significant increment in the total cytoplasmic levels of keap1, which is a natural inhibitor of Nrf2 that stimulates its cytoplasmic proteasomal degradation. This occurred even without any alterations in the mRNA levels of keap1. These data suggest that hyperglycemia inhibits the degradation of keap1 and is a major mechanism for reducing the activation of Nrf2. These data support other in vivo and in vitro studies that reported similar effects [13,17,18,19,20,21]. Opposing this, treatment with ESA stimulated the total cytoplasmic and nuclear localization of Nrf2 and reduced the cytoplasmic levels of keap1 in the LVs of the STZ-diabetic rats. It also enhanced the cytoplasmic and nuclear levels of Nrf2 in the LVs of the control rats with no alteration in the mRNA level of this factor. ESA also does not affect the mRNA levels of keap1 in any group of rats. Even though ESA could stimulate Nrf2 by reducing cardiomyocyte apoptosis, the observation obtained from this study suggests that ESA enhances the transactivation of Nrf2 through the hyperglycemia-independent-induced degradation of keap1 in the cytoplasm. It could be also possible that ESA stimulates Nrf2 by modifying the cysteine residues of keap1. Indeed, several Nrf2-activating plant-derived drugs act by forming adducts with the cysteine residues or stimulating the degradation of kepa1 [68]. However, all of the cardiac protective effects afforded by ESA on cardiac hemodynamics, structure, antioxidant levels, and suppression of lipid peroxidation, inflammation, fibrosis, and apoptosis were prevented by brusatol. Based on these data, we become very confident that ESA is a potent activator of Nrf2 signaling, which inhibits the interaction between Nrf2 and keap1, possibly by modifying the keap1 cysteine residues needed to bind Nrf2. This suggestion cannot be confirmed from the data of this study and needs further precise examination. Nevertheless, such activation of Nrf2 signaling by ESA explains why the LVs of the control and diabetic hearts had higher levels of GSH, antioxidant enzymes, and Bcl2. It also indicates that the inhibitory effect of ESA on NF-κB and TGF-β could occur directly through the activation of Nrf2 or indirectly through suppressing oxidative stress and apoptosis. 

However, the precise mechanism by which ESA affords these cardioprotective effects cannot be depicted in this study and could be considered a major limitation. At this stage, we suggest that the effect of ESA over all other glycosides is related to its direct effect due to its unique structure or due to generating diverse metabolites after oral ingestion. In general, the oral administration of most steroidal glycosides yields metabolite derivatives of pregnane, which is a type of steroidal hormone [69]. In addition, they generate metabolites with hydroxyl groups that can also act within the cell to regulate cell signaling and oxidative stress. An example is the metabolism of the spirostane, spirosolane, and furostane steroid glycosides, leading to the formation of a hydroxyl group at C-23 and that is metabolized into pregnane after oral ingestion [69]. Here, we expect that the ESA is metabolized in a similar way to exert these interesting biological effects. Yet, determining the number and locations of these hydroxyl groups needs further examination. In addition, the intestinal metabolism of ESA has not been established yet and needs further clarification to explain its cardioprotective effect and its role in improving hyperlipidemia, suppressing cardiac NF-κB and ROS, and activating Nrf2. In addition, recent studies have shown the unique structure of ESA, which makes it different to other glycosides (Nohara), that is, C58H95NO29 ((23 S, 25 S)–23–acetoxy–5 α, 22 α N–3β, 27–dihydroxyspirosolan 3–O–β–lycotetraosyl 27–O–β–D–glucopyranoside) [69]. There are 10 free hydroxyl radicals within this structure, which may make ESA a potent antioxidant molecule over all other glycosides. 

## 5. Conclusions

The data presented in this work identify ESA as a novel molecule that can alleviate diabetic cardiomyopathy in STZ-T1DM rats. Based on the evidence presented here, it seems that ESA acts mainly by exerting antioxidant and anti-inflammatory effects. In addition, activating Nrf2 by suppressing keap1 is the one mechanism of action. However, patients with HF may also develop T2DM due to the reduced circulation and secretion of insulin. Based on our data, as ESA has no effects on glucose and insulin levels, it seems reasonable that ESA could utilize cardiac-related mechanisms to activate the Nrf2/antioxidant axis. These data are very interesting and may suggest further clinical trials in humans. In addition, they open a window to study the cardioprotective effect of this molecule in any other model where the activation of Nrf2 is a reported therapeutic strategy.

## Figures and Tables

**Table 1:** Primers used in the q-PCR reaction.

Target	Primers Sequence 5′→3′	Accession No.	Base Pair Length
Nrf2	F:AAAATCATTAACCTCCCTGTTGAT R:CGGCGACTTTATTCTTACCTCTC	NM_031789	118
NF-κB	F:GTGCAGAAAGAAGACATTGAGGTG R:AGGCTAGGGTCAGCGTATGG	XM_342346.4	176
Keap1	F:CTTCGGGGAGGA GGAGTTCT R:CGTTCAGATCATCGCGGCTG	NM_057152.2	122
β-actin	F:CGAGTACAACCTTCTTGCAGC R:CCTTCTGACCCATACCCACC	NM_031144.3	209

**Table 2:** Final body and heart weights and levels of cardiac function markers in all groups of rats.

Parameter	Control	ESA	STZ-T1DM	STZ-T1DM + ESA	DM + ESA + Brusatol
Final body weight (g)	523.2 ± 43.2	541.1 ± 44.6	346.2 ± 28.4 ab	463.3 ± 22.9 abc	331.3 ± 34.1 abd
Heart weight (g)	1.68 ± 0.25	1.59 ± 0.24	2.23 ± 0.21 ab	1.61 ± 0.11 c	2.17 ± 0.21 abd
LVSP (mmHg)	117.1 ± 10.2	112.8 ± 11.4	73.4 ± 6.5 ab	103.3 ± 9.7 c	69.8 ± 7.6 abd
LVEDP (mmHg)	2.75 ± 0.36	3.11 ± 0.37	12.36 ± 1.4 ab	4.43 ± 0.56 abc	11.46 ± 1.6 abd
Dp/dt_max_ (×10^3^ mmHg)	6.74 ± 0.54	6.29 ± 0.73	3.46 ± 0.43 ab	5.89 ± 0.73 c	3.18 ± 0.36 abd
Dp/dt_min_ (×10^3^ mmHg)	5.43 ± 0.63	5.88 ± 0.49	2.73 ± 0.22 ab	5.17 ± 0.63 c	2.39 ± 0.26 abd
TpI (pg/mL)	24.5 ± 2.7	22.5 ± 2.1	127.2 ± 11.5 ab	27.8 ± 3.9 bc	136.3 ± 15.2 abd
CK-MB (IU/L)	16.4 ± 2.6	15.8 ± 2.4	85.4 ± 7.9 ab	18.8 ± 2.9 c	91.2 ± 10.4 abd
LDH (IU/mL)	229.2 ± 29.8	231.7 ± 18.3	387.3 ± 42.3 ab	247.2 ± 33.1 c	415.6 ± 52.4 abd

Data are presented as mean ± SD. Level of significance = *p* < 0.05. a: significant vs. control group. b: significant vs. ESA (100 mg/kg)-treated rats, c: significant vs. STZ-T1DM model group. d: significant vs. different STZ-T1DM + ESA (100 mg/kg)-treated rats. ESA: Esculeoside A (ESA). Brusatol: an Nrf2 inhibitor.

**Table 3:** Effect of all treatments on fasting plasma glucose and insulin levels, as well as on serum lipid profile in all groups of rats.

Parameter	Control	ESA	STZ-DM	STZ-DM + ESA	STZ-DM + ESA + Brusatol
FPG (mg/dL)	113.5 ± 10.4	108.9 ± 10.2	328.4 ± 44.7 ab	318.4 ± 28.5 ab	341.2 ± 38.1 ab
FPI (µIU/mL)	3.23 ± 0.42	3.59 ± 0.33	1.63 ± 0.19 ab	1.52 ± 0.27 ab	1.48 ± 0.20 ab
TGs (mg/dL)	74.4 ± 8.1	61.2 ± 8.4 a	167.1 ± 15.9 ab	92.3 ± 8.4 abc	181.2 ± 22.3 abd
CHOL (mg/dL)	84.6 ± 7.4	68.5 ± 7.6 a	182.3 ± 15.2 ab	81.2 ± 8.3 bc	167.3 ± 17.4 abd
LDL-c (mg/dL)	36.7 ± 3.1	24.5 ± 2.5 a	87.9 ± 7.7 ab	35.9 ± 4.2 bc	92.3 ± 10.3 abd

Data are presented as mean ± SD. Level of significance = *p* < 0.05. a: significant vs. control group. b: significant vs. ESA (100 mg/kg)-treated rats, c: significant vs. STZ-T1DM model group. d: significant vs. different STZ-T1DM + ESA (100 mg/kg)-treated rats. ESA: Esculeoside A (ESA). Brusatol: an Nrf2 inhibitor.

**Table 4:** Effect of all treatments on selected markers of oxidative stress and inflammation in the left ventricles (LV) of all groups of rats.

Parameter	Control	ESA	STZ-DM	STZ-DM + ESA	STZ-DM + ESA + Brusatol
MDA (nmol/mg tissue)	0.53 ± 0.08	0.63 ± 0.072	1.72 ± 0.21 ab	0.61 ± 0.09 c	1.68 ± 0.15 ab
GSH (nmol/mg tissue)	63.4 ± 7.3	78.4 ± 6.4 a	36.6 ± 4.1 ab	65.2 ± 6.4 bc	31.1 ± 2.9 ab
HO-1 (pg/mg tissue)	20.4 ± 3.1	32.8 ± 3.3 a	11.3 ± 2.2 ab	26.5 ± 2.8 bc	9.4 ± 1.3 abd
SOD1 (pg/mg tissue)	28.9 ± 2.7	41.8 ± 3.7 a	12.9 ± 1.5 ab	26.8 ± 2.2 bc	10.6 ± 1.1 abd
TNFα (pg/mg tissue)	0.89 ± 0.06	1.1 ± 0.13	4.93 ± 0.82 ab	1.27 ± 0.16 ac	5.16 ± 0.08 abd
ICAM-1	54.6 ± 6.9	59.8 ± 5.2	134.5 ± 12.9 ab	57.4 ± 6.6 c	126.3 ± 14.5 abd
IL-6 (pg/mg tissue)	2.89 ± 0.31	3.25 ± 0.58	13.22 ± 1.82 ab	4.22 ± 0.72 abc	11.6 ± 1.42 abd

Data are presented as mean ± SD. Level of significance = *p* < 0.05. a: significant vs. control group. b: significant vs. ESA (100 mg/kg)-treated rats, c: significant vs. STZ-T1DM model group. d: significant vs. different STZ-T1DM + ESA (100 mg/kg)-treated rats. ESA: Esculeoside A (ESA). Brusatol: an Nrf2 inhibitor.

## Data Availability

The datasets used and analyzed in the current study are available from the corresponding author upon reasonable request.
